# The influence of removing home advantage on the Chinese Football Super League

**DOI:** 10.1186/s13102-022-00604-0

**Published:** 2022-12-09

**Authors:** Bo Han, Lang Yang, Pengyu Pan, Antonio García-de-Alcaraz, Can Yang, Tianbiao Liu

**Affiliations:** 1 China Football College, Beijing Sport University, Beijing, 100084 China; 2 Present Address: Beijing Zhongguancun Foreign Language School, Beijing, 100085 China; 3 College of Physical Education and Sports, Beijing Normal University, Beijing, 100875 China; 4 Department of Education, Faculty of Education Sciences, University of Almería, Almería, Spain; 5 SPORT Research Group (CTS-1024), CERNEP Research Center, University of Almería, Almería, Spain

**Keywords:** Team sports, Performance analysis, Situational variables, Performance indicators, Predictive statistic, Home advantage, Match location, Neutral venue

## Abstract

**Background:**

Due to the COVID-19 pandemic, the 2020 season Chinese Super League (CSL) was held in neutral venues, this study aims to analyse the impact of removing home advantage (HA) in CSL.

**Method:**

240 games of the CSL 2019 season (home and away double round-robin system) and 160 games of the 2020 season (in neutral venues) were analysed. 27 technical and tactical performance indicators were involved as dependent variables. A multiple linear regression model was established to analyse the influence of removing HA on the performance indicators.

**Results:**

After moving from home stadium to neutral venue in 2020 season, goal, shot, shot on target, shot from outside box, shot from inside box, shot on target from inside box, corner kick, key pass, cross, breakthrough, tackle decreased significantly (*p* < 0.05), while yellow card and foul increased steeply (*p* < 0.05). Comparing with playing away match, in neutral venue, free kicks and pass accuracy enhanced radically (*p* < 0.05), while tackle, clearance and block shot dropped noticeably (*p* < 0.05).

**Conclusion:**

When removing HA and playing in the neutral venue, teams' performance dropped significantly. This study confirmed the positive impact of HA on the teams' performance and may help elite football teams make proper playing strategies regarding different match locations.

## Introduction

Home advantage (HA) is one of the most important factors affecting the outcome of the game [1, 2]. Previous studies found that presence of HA in various sports, especially in indoor compared to outdoor ones. For example, ice hockey and basketball have a higher HA than American football and baseball [1, 3]. When a country hosted the Olympic Games, the athletes’ performance from host country would also improve [4, 5], and the host country of the Winter Olympics had a greater HA in men’s competitions than the Summer Olympics [6]. Moreover, there is a HA in both individual sports [7] and team sports [8]. In team sports, this phenomena is greater in men's competitions, especially in football [9] and water polo [10]. Furthermore, after comparing different levels of leagues, it is found that lower-level leagues often have a greater HA than higher-level leagues [10, 11].

The factors affecting HA mainly refer to fans and crowd support, familiarity with the venue, travel conditions and referee bias. The behaviour of the spectators on the stands will influence the referee's psychology, causing bias in the referee's decision [12] and making the referee unconsciously favour the home team [13–15]. At the same time, the support of the fans also helps to improve the performance of the home team [16]. Familiarity with the game environment, such as familiarity and adaptation to the field conditions, environmental features, lighting, temperature, humidity, etc., would also have a positive impact on the home team [17]. The long-distance travel would have a negative impact on the away team, leading to poor performance in the game [15, 18, 19]. Therefore, the existence of HA can give underdogs a chance to defeat the strong team at home [20].

HA was also found in the Chinese professional football league in previous research. During 1994–1996 season, the probability of undefeated at home for the Jia A (1st division league) and Jia B (2nd division league) teams was more than 70% [21]. Before the 2004 season, the home winning ratio of the Jia A (Jia A was renamed Chinese Super league in 2004) was 47% [22] and Pollard and Gómez [9] reported a HA of 63.82% through the 2006–2012 seasons. In addition, a recent study found that the average HA of the CSL in the 2014–2016 season was 59.7% and 72.78% of the home teams remained unbeaten [23]. Although the HA in the CSL kept reducing in the past 20 seasons, the home teams still gained more league points and were approximately 13.42% more likely to win than away teams (data from 2014 to 2018 season) [24]. Previous studies found that match location (ML) and team strength (TS, whether a team is superior or inferior) as well as quality of opposition (QO, whether a team’s opponent is superior or inferior) were important situational variables affecting the team’s technical and tactical performance [25, 26]. Home team’s offence related indicators were higher than away team [23, 26, 27]. The connection between the players of the home team was better and the success rate of group work was higher [8]. However, HA would be also influenced by the strength of the team [28]. Strong teams play better at home and away. Although weak teams still lose to strong teams at home, they would have better key performance indicators and defeat their rivals of the same or similar level at their home stadiums [29]. Therefore, it is also necessary to consider other factors surrounding ML such as team strength (TS) and quality of opposition (QO) in the study of HA.

The outbreak of the COVID-19 pandemic had a significant impact on global sport events. A large number of sport events were suspended or held in empty stadiums. European professional football leagues retained home and away double round-robin system after the restoration, but the fans were not allowed to enter the stadiums in some games. Some studies were conducted from the perspective of crowd support [30]. After the social distancing by COVID-19, the HA of German Bundesliga dropped from 50.32 to 40.37% [31]. Research about 15 leagues in 11 European countries found HA (goals scored and points gained) was significantly reduced during- COVID (no crowd) and home teams created significantly fewer attacking opportunities [32]. Results regarding Brazilian elite football also indicated that HA was reduced in Serie A in 2020 (absence of the crowd), but no changes in HA in Serie B was found [33]. These might be because empty stadiums reduced the pressure on the referees [34] and decreased the referee bias to home teams [35]. Referees gave more fouls against the home team and less yellow and red cards to the away team when the audience was absent in European leagues [32].

Due to the impact of the pandemic, the 2020 CSL changed the competition system. The new competition system comprised of two stages. In the first stage, based on the rank of the 2019 CSL, the 16 teams were divided into two groups, with eight teams in each group (Group A and Group B). The competitions of Group A were held in Dalian (A city in north-eastern China) and Group B in Suzhou (A city in eastern China). A total of 14-rounds matches (double round-robin system) were completed in each group. After the first stage, the top four of each group advanced to the "Champion Group", with a total of eight teams; the bottom four of each group moved into the "Relegation Group". In the second stage, the eight teams in "Champion Group" competed through two rounds of knockout matches until the champion emerged; the eight teams in "Relegation Group" played in the same way until the two relegated teams came into being. Compared with 2019 season, 2020 season CSL under the new competition system deceased the number of games from 240 to 160 matches and all games in 2020 season were played in neural venues without fans. Hence, in addition to the fact that the participating teams in the 2020 season CSL were immune to spectator factors, the HA related factors such as familiarity with the stadium and long-distance travel no longer existed. Some studies compared the difference between the two stages (before and after the suspension) of the same season [36], using unbalanced opponent samples (home-away/strong–weak). In comparison, this current study compared the technical and tactical performance of the CSL teams of two complete seasons (before and after the pandemic suspension). Thus, this study aimed to analyse the impact of removing HA in CSL, which can provide reference and support for the teams to formulate relevant game strategies for different match locations and competition systems.

## Materials and methods

### Sample and performance indicators

The sample comprised 400 matches of CSL during 2019 and 2020 seasons, including 240 matches in the 2019 season (home and away double round-robin system) and 160 games in the 2020 season (played in neutral venues of two cities due to the COVID-19 pandemic). Based on previous studies [23], a total of 27 technical and tactical indicators, as well as three situational variables (match location, team strength and quality of opposition) were analysed (Table 1).

**Table 1:** Match-related performance indicators

*Goals scored performance-related parameters: operational definition*	
Goal	The number of times a goal has been scored when the entire ball has crossed the goal line, between the goal posts and under the crossbar, provided the shooting team has not committed a foul
Penalty	Player fouled within the penalty box leading to a penalty kick
Shot	An attempt to score a goal, made with any (legal) part of the body, either on or off target
Shot on target	An attempt to score a goal, which required intervention to stop the ball going in or resulted in a goal/shot that would have gone in without diversion
Shot from outside box	A shot from outside the penalty area
Shot on target from outside box	A shot on target from outside the penalty area
Shot from inside box	A shot inside the penalty area
Shot on target from inside box	A shot on target inside the penalty area
Free kick	Number of free kicks awarded
Free kick in front field	Number of free kicks awarded on the opponent's half of the pitch
Corner kick	Ball goes out of play for a corner kick
Breakthrough	The number of times the ball handler successfully got rid of an opposing player after dribbling past that player
*Offence performance-related parameters: operational definition*	
Possession (%)	The duration when a team takes over the ball from the opposing team without any clear interruption as a proportion of total duration when the ball was in play
Pass	An intentional played ball from one player to another
Pass accuracy (%)	Successful passes as a proportion of the total passes
Key pass	The final pass assisting a shot (without scoring)
Pass in the attacking third	Number of passes of the ball (possessed by the attacking team) in the 35 m area of the opponent's half of the pitch
Pass accuracy (%) in the attacking third	Number of successful passes of the ball (possessed by the attacking team) as a proportion of the total passes in the 35 m area of the opponent's half of the pitch
Cross	Balls sent into the central area of the box from a wide position of the attacking third
*Defence performance-related parameters: operational definition*	
Tackle	The action of gaining possession from an opposition player who is in possession of the ball
Interception	A player intercepts a pass between oppositions and prevents the opponent receiving the ball
Clearance	A player kicks or hits the ball away from the goal of his or her own team without a precise target
Foul	Any infringement that is penalised as foul play by a referee
Yellow card	A player is shown a yellow card by the referee
Red card	A player is sanctioned a red card by the referee
Block pass	Number of blocked passes completed
Block shot	Number of blocked shots completed
*Situational Variables performance-related parameters: operational definition*	
Match location (ML)	The competition venue is for the participating teams to play the game, which is divided into home, away and neutral
Team strength (TS)	Strength of the participating team. TS is evaluated using standardized team market value by the beginning of the season. 16 teams’ market value was converted into the relative strength of the team using the standard score (Z-score) in statistics
Quality of opposition (QO)	Strength of the opponent. QO is evaluated using standardized team market value by the beginning of the season. 16 teams’ market value was converted into the relative strength of the team using the standard score (Z-score) in statistics

### Data source and reliability

The technical and tactical data used in this research came from Champdas Football Big Data Company (http://www.champdas.com). The reliability and validity of the Champdas Football Data collection and analysis system had been validated by other researchers [37]. In addition, the team strength in this study did not use the end-of-season rankings, because the end-of-season rankings are the result of the performance of the team in the current season, in order to avoid the influence of endogenous variables, the pre-season market value of the team is selected to measure the team strength. Studies have shown that the total player value of a club is one of the important factors that positively affects the overall strength of the club [38–40]. Therefore, current study used the pre-season market value of CSL teams to measure the team strength according to previous research [24, 29] and consequently the total value of the team was standardized and ranked when calculating the team strength. The data of team market value came from the website “Transfermarkt” (http://www.transfermarkt.de) and the data from this website have been used in previous research [24, 29, 40, 41].

### Procedures and statistical analysis

All data were organized via Excel and imported into R Studio for pre-processing and data cleaning. Afterwards, an independent sample T-test (Mean ± SD) was conducted to explore the differences among home team performance and away team performance in the 2019 season and all team performance in the 2020 season. Consequently, match location (ML) was divided into three situations: home and away in the 2019 season and all games in the 2020 season. The 27 technical and tactical performance indicators (PI) acted as the dependent variable and the match location (ML) worked as the independent variable. In addition, the team strength (TS) and quality of opposition (QO) were included as the covariates. The model firstly selected the home games in the 2019 season as the baseline. A multiple linear regression model was established:

1 $${PI}_{i}={\beta }_{0i}+\sum_{i=1}^{2}{\beta }_{1i}\cdot ML+{\beta }_{2i}\cdot TS+{\beta }_{3i}\cdot QO+{\varepsilon }_{i}$$

Moreover, several indicators including penalty, yellow card and red card are subject to Poisson distribution, which have been studied in previous research [42]. Thus, the present study selected generalised liner model for these indicators. Each model’s homogeneity and normal distribution assumptions were confirmed with no particular issues and the validity of each model has been tested and verified. All analyses used the lmerTest [43] package in the R (ver.4.1.1) (R Core Team, 2021) statistical software and the significance level was set to α = 0.05.

## Results

**Fig. 1:**
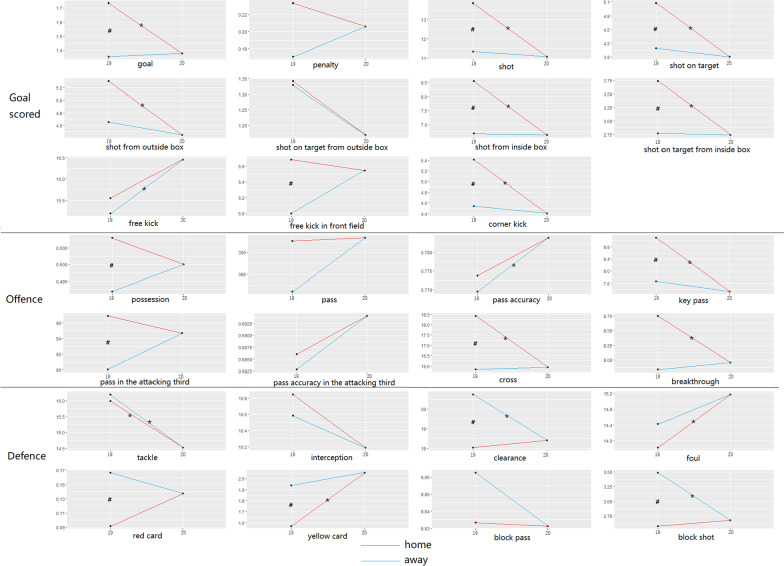
Comparison of home and away teams’ performance indicators between the 2019 and 2020 season CSL. ^#^Significant difference between home and away in 2019 season (*p* < 0.05). *Significant difference between 2019 and 2020 season (*p* < 0.05)

The difference of home and away teams’ performance indicators between the 2019 season and 2020 season CSL are shown in Fig. 1. After moving from home stadium to the neutral venue, goal, shot, shot on target, shot from outside box, shot from inside box, shot on target from inside box, corner kick, key pass, cross, breakthrough, tackle decreased significantly (*p* < 0.05), while yellow card and foul increased steeply (*p* < 0.05). Comparing with away matches in 2019 season, when teams played in the neutral venues in 2020 season, free kicks and pass accuracy enhanced radically (p < 0.05), while defending performance including tackle, clearance and block shot dropped noticeably (p < 0.05).

Table 2 illustrates the results of the multiple linear regression model. The results show that ML has a significant impact on the performance indicators of the game. Meanwhile, TS and QO also influenced the performance indicators together with ML. When the home games in the 2019 season are set as the baseline, such goal related indicators in the 2020 season (in the neutral venues) as goals, shots, shots on target, shots from outside the box, shots from inside the box, shots on target from inside the box, corner kicks all had significant negative effect, but the free kicks had a significant positive effect, compared with home games in the 2019 season. Specifically, the average goals dropped by 0.355 (*p* < 0.001) and overall shots went down by 2.781 (p < 0.001). Shots consists of shots from both inside or outside the box, which both also fell off. Shots on target (-1.166, *p* < 0.001) and shots from inside the box (− 1.928, *p* < 0.001) declined by a higher proportion. In addition, corner kicks dropped by 1.006 (*p* < 0.001), whereas free kicks per game increased by 0.910 (*p* < 0.05). Amongst the offence related indicators, key passes and crosses produced significant negative effects compared to home games in the 2019 season. Key passes per game decreased by 1.769 (*p* < 0.001) and crosses per game decreased by 2.470 (*p* < 0.001), while the pass accuracy (%) having a significant positive effect increased by 0.010 (*p* < 0.05). Amongst the defence related indicators, tackles per game decreased by 1.480 (*p* < 0.001), compared to the home games in the 2019 season, whereas fouls increased by 1.347 (*p* < 0.001) and yellow cards mount up by 0.487 (*p* < 0.001).

**Table 2:** Results of the multiple linear regression model

Indicators	2019 season home	Fixed intercept	2019 season away	2020 season neutral venue	Team strength	Quality of opponent	F test (p)
Goal	Baseline	1.733***	− 0.379***	− 0.355***	0.303***	− 0.281***	< 0.001
Penalty		0.224***	− 0.050	− 0.014	0.013	− 0.047**	0.022
Shot		13.850***	− 2.521***	− 2.781***	1.241***	− 0.656***	< 0.001
Shot on target		5.075***	− 0.979***	− 1.166***	0.659***	− 0.466***	< 0.001
SfOB		5.300***	− 0.650**	− 0.853***	0.360***	0.031	< 0.001
STfOB		1.342***	− 0.013	− 0.173	0.216***	− 0.031	< 0.001
SfIB		8.550***	− 1.871***	− 1.928***	0.880***	− 0.687***	< 0.001
STfIB		3.733***	− 0.967***	− 0.993***	0.443***	− 0.435***	< 0.001
FK		15.546***	− 0.354	0.910*	− 0.519***	0.040	< 0.001
FKFF		6.688***	− 0.688**	− 0.141	− 0.181	− 0.307**	< 0.001
Corner		5.413***	− 0.871***	− 1.006***	0.393***	− 0.218*	< 0.001
Possession (%)		0.508***	− 0.016**	− 0.008	0.025***	− 0.025***	< 0.001
Pass		392.633***	− 11.546	0.746	41.022***	− 23.532***	< 0.001
Pass accuracy (%)		0.774***	− 0.004	0.010*	0.021***	− 0.009***	< 0.001
Key pass		9.100***	− 1.438***	− 1.769***	0.837***	− 0.348**	< 0.001
PAT		96.862***	− 6.808*	− 2.187	13.752***	− 6.481***	< 0.001
Pass accuracy (%) AT		0.686***	− 0.003	0.008	0.022***	− 0.005*	< 0.001
Cross		18.417***	− 2.583***	− 2.470***	0.880***	− 0.843***	< 0.001
Breakthrough		8.746***	− 0.904	− 0.783	0.413*	0.282	0.016
Tackle		15.996***	0.208	− 1.480***	0.800***	0.296	< 0.001
Interception		10.846***	− 0.263	− 0.658	0.397*	0.873***	< 0.001
Clearance		18.025***	2.738***	0.384	− 0.726*	0.606*	< 0.001
Foul		13.825***	0.604	1.347***	− 0.170	− 0.428**	< 0.001
Yellow card		1.567***	0.375**	0.487***	− 0.085	0.081	< 0.001
Red card		0.104***	0.058	0.029	0.003	− 0.033**	0.011
Block pass		8.833***	0.029	− 0.002	− 0.107	0.445***	0.015
Block shot		2.579***	0.908***	0.096	− 0.034	0.109	< 0.001

*SfOB*: Shot from outside the box, *STfOB*: Shot on target from outside box, *SfIB*: Shot from inside the box, *STfIB*: Shot on target from inside the box, *FK*: Free kick, *FKFF*: Free kick in front field, *PAT*: Pass in the attacking third, *Pass accuracy (%) AT*: Pass accuracy (%) in the attacking third

Significance level ***p < 0.001, **p < 0.01, *p < 0.05

## Discussion

The purpose of this study was to explore the impact of removing HA on the performance of CSL teams. This research found that after moving from home stadium to neutral venues in 2020 season, CSL teams’ performance reduced, especially in goals scoring and offence performance indicators. Comparing with away matches in 2019 season, teams playing in the neutral venues in 2020 season showed similar performance with visiting teams.

The COVID-19 pandemic affected professional football leagues in almost all countries around the world in 2020. Thus, the competition system of CSL in the 2020 season was changed to a new system due to the pandemic and the games were played without fans in neutral venues. The change of the competition system enabled all teams in the 2020 season to avoid crowd support and long-distance travel and all teams were identically familiar to the stadiums. From the perspective of scientific research, this is a more ideal experimental environment without any HA effect. The results of research on the German Bundesliga, English Premier League, Spanish La Liga, French Primeira Liga, Italian Serie A and other leagues during the pandemic showed that the home teams’ shots and shots on target significantly reduced and the number of fouls and yellow cards increased significantly [16, 44–50]. These findings are consistent with the results of current study. Some studies found that the supporters’ shouts and cheers from the stands of the stadium can increase the home players’ testosterone level, so that the players would be more aggressive and motivated at home [51]. The COVID-19 pandemic resulted in empty stadium play, perhaps due to lack of support from home fans in the stadium, the players' behaviours and actions related to goals scoring declined and the home team players’ desire for offensive performance decreased [31, 50]. Combining with the results of multiple linear regression, the results found that all teams of CSL were equivalent to playing “away games” in the 2020 season (in neutral venues).

The away teams pass accuracy (%) improved greatly in the neutral venues performed in the 2020 season in the CSL. This indicated the away teams had more time and space for making successful passes, which enhanced pass accuracy (%). Meanwhile, the away team's defensive indicators declined, for instance, tackles, clearances and block shots. This might be related to the home teams’ decline in offensive performance. Thus, if the home team's shots and shots on target decreased significantly, then the away team's block shots would probably fall as well. Before the pandemic, some studies found that when teams played at home, their number of passes and pass accuracy (%) were higher [25, 46]. After that, the game data in empty stadiums showed that lack of support from the fans did not cause the decrease of home teams' pass accuracy (%) [31, 50]. Perhaps in the empty stadiums, the players' mentality in the attack might be more stable and they would prefer to make safer and low- risk passes. At the same time, it may be related to the fact that coaches could provide more effective verbal guidance and feedback to players on the field in the quiet stadium, which would make players' behaviours more rational [31, 51].

Compared with the 2019 season, both home and away teams’ free kicks in the 2020 season were on the rise. Before the pandemic, Liu, García-de-Alcaraz [23] found that the teams were awarded less free kicks at home than away. Current study found that the number of free kicks given to the home team increased significantly in the 2020 season. However, there was no significant increase in the number of free kicks in the front field. This implied that home teams in neutral venues got more free kicks in their own half field. This could be probably caused by the opponent's offside, or it might be a result from the opponent's aggressive high press. At the same time, the away teams’ free kicks in the front field increased significantly in the neutral stadiums. This may be due to the rise of away teams’ offensive performance in the neutral stadiums, which caused more threats to the home teams and the home teams committed more fouls [34].

Previous study found that away teams of CSL were given more yellow cards [23], which was also consistent with the results of current study. In the 2020 season, when HA disappeared, the home teams' yellow cards increased by 0.48 per game. However, it is worth noting that the away teams’ yellow cards also increased slightly in the 2020 season. Leitner and Richlan [34] compared the leagues of eight European countries during the pandemic and found that in empty stadiums, the home teams’ yellow cards also increased significantly. The reasons for yellow cards could be criticism, unfair sportsmanship and fouls. After comparing the three categories of yellow cards, it is found that in empty stadiums, yellow cards awarded for fouls increased strongly for home teams by 26.2% but only slightly for away teams (+ 2.8%). A study of the Bundesliga discovered that the number of yellow cards without fans was significantly higher than when there were fans in the stadiums before the pandemic [49, 52–54]. This might be because the referees were under less pressure from the supporters in an empty stadium, they were more determined when giving a yellow card.

Last but not the least, current study did not find the contribution of long travel and familiarity to HA. In the 2020 season, teams were playing in one city and didn’t need to travel long distance. It was believed that long distance travel was significantly related to goals scored (r = − 0.08, *p* < 0.001), which means that teams scored fewer goals as they travel longer distance [19]. This result is not consistent with current study because the away teams’ goals scored in the 2020 season CSL did not increase although they did not need to travel. Therefore, it seems that the travel fatigue in CSL had little influence on away teams’ goals scored. Moreover, regarding the relations between facility familiarity and HA, previous research found that “special” fields (larger, smaller or artificial turf) may bring very subtle advantage to home team [18]. In current study, no evidence could be discovered to support that stadium familiarity would contribute to HA in CSL and familiarity remains a plausible but unsubstantiated factor affecting HA. In addition, this study has limitations in using transfermarket data to measure team strength, because players’ value is influenced by many factors, such as current age, future skill, value of previous years and so on. Thus, teams that appear to have more value may not actually have better team strength for the current season. In future research, models from other researchers could be considered to better solve this problem. This study sheds light on the HA from a new perspective, exploring the changes of technical and tactical indicators when the HA is completely removed, however, HA is a multifactorial phenomenon, various data and factors should be put into consideration, such as fitness data and the modification of football law (number of substitutions, yellow or red cards to the team officials and etc.). Further studies may seek to detect the influence of removing HA in different countries and collect more diversified data to analyse their relations to HA.

## Conclusions

When HA is removed, CSL teams’ performance decreased in the neutral venues. Compared with the away games in the 2019 season, the performance of teams in the neutral venues (2020 season) did not change much. Therefore, playing in neutral venues is equivalent to play away games for all the teams.

In the CSL, ML has a significant impact on teams’ performance. The results of the data analysis also confirmed that the team strength, quality of opposition and ML affected the performance of the teams. This study verified the positive impact of HA on teams’ performance. Therefore, professional football clubs or national teams can improve their performance at home by actively hosting football events, attracting more fans to the stadium to cheer for the team.

## Data Availability

The data that support the findings of this study are available from Champdas Football Big Data Company (http://www.champdas.com), but restrictions apply to the availability of these data, which were used under license for the current study, and so are not publicly available. Data are however available from the authors upon reasonable request and with permission of Champdas Football Big Data Company.

## Abbreviations

CSL: The Chinese Football Super League
HA: Home advantage
PI: Performance indicator
ML: Match location
TS: Team strength
QO: Quality of opposition
